# Long Non-coding RNA BGas Regulates the Cystic Fibrosis Transmembrane Conductance Regulator

**DOI:** 10.1038/mt.2016.112

**Published:** 2016-07-19

**Authors:** Sheena M Saayman, Amanda Ackley, Jon Burdach, Matthew Clemson, Dieter C Gruenert, Kiyoshi Tachikawa, Pad Chivukula, Marc S Weinberg, Kevin V Morris

**Affiliations:** 1 Molecular and Experimental Medicine, The Scripps Research Institute, La Jolla, California, USA; 2 Biotechnology and Biomedical Sciences, The University of New South Wales, Sydney, Australia; 3 Department of Otolaryngology-Head and Neck Surgery, Eli and Edythe Broad Center for Regenerative Medicine and Stem Cell Research, Institute for Human Genetics, The University of California San Francisco, San Francisco, California, USA; 4 Arcturus, Therapeutics, San Diego, California, USA; 5 Wits/SAMRC Antiviral Gene Therapy Research Unit, School of Pathology, University of the Witwatersrand, WITS 2050, South Africa; 6 HIV Pathogenesis Research Unit, Department of Molecular Medicine and Haematology, School of Pathology, University of the Witwatersrand, WITS 2050, South Africa; 7 City of Hope – Beckman Research Institute, Center for Gene Therapy, Duarte, California, USA

## Abstract

Cystic fibrosis (CF) is a life-shortening genetic disease. The root cause of CF is heritable recessive mutations that affect the cystic fibrosis transmembrance conductance regulator (*CFTR*) gene and the subsequent expression and activity of encoded ion channels at the cell surface. We show that *CFTR* is regulated transcriptionally by the actions of a novel long noncoding RNA (lncRNA), designated as BGas, that emanates from intron 11 of the *CFTR* gene and is expressed in the antisense orientation relative to the protein coding sense strand. We find that BGas functions in concert with several proteins including HMGA1, HMGB1, and WIBG to modulate the local chromatin and DNA architecture of intron 11 of the *CFTR* gene and thereby affects transcription. Suppression of BGas or its associated proteins results in a gain of both *CFTR* expression and chloride ion function. The observations described here highlight a previously underappreciated mechanism of transcriptional control and suggest that BGas may serve as a therapeutic target for specifically activating expression of *CFTR*.

## Introduction

Cystic fibrosis (CF) is an autosomal recessive disease that arises as a result of defects in the CF transmembrane conductance regulator (*CFTR*) gene^1,2,3^ that encodes an ion channel in the apical membrane of epithelial cells. Approximately 2,000 mutations in the *CFTR* gene have been identified to date. *CFTR* variants have been largely categorized into six classes according to their effect on CFTR and the resulting phenotype. Phenotypes may include low CFTR protein levels, low CFTR protein localization at the cell surface and channel activity deficiencies.^4^ The genotype-phenotype relationship of many of these mutations, however, is yet to be characterized. Furthermore, the degree of disease manifestation in CF patients is highly variable, yet the genotype of affected individuals does not always correlate with clinical severity.^5^ The lack of correlation suggests that although CF is monogenic, it is a multifaceted complex disorder with multiple contributing factors. Recently, genome-wide association analysis identified five modifier genes that contributed to lung disease in CF patients.^6^ In addition, epigenetics has also been shown to be a contributing factor in CF disease variability.^7^ The regulatory mechanisms governing *CFTR* expression are complex and are still not entirely understood. It is evident, however, that histone modifications and DNA methylation may play a role in *CFTR* expression, suggesting an epigenetic component to *CFTR* transcriptional regulation. Furthermore, histone deacetylase (HDAC) inhibitors have been shown to partially restore the DeltaF508 mutant phenotype in human primary airway epithelia, which signifies the potential for epigenetic therapies.^8,9^

An emerging body of evidence suggests that endogenous long noncoding RNAs (lncRNAs) are involved in epigenetically regulating gene expression in human cells (reviewed in refs. 10,11). Long noncoding RNAs are extremely diverse with respect to their transcriptional origins as well as their mechanisms of action, and may also be expressed in the sense or antisense orientation relative to their protein-coding gene counterparts.^12^ Several lncRNAs, that function in the target specific recruitment of epigenetic complexes and transcriptional silencing have been identified.^13,14^ However, little is known about those lncRNAs involved in monoalleic disease, such as CF. We identify here a lncRNA associated with the *CFTR* gene and determine its mechanistic role in the regulation of *CFTR* transcription. We report here that this lncRNA functions to modulate *CFTR* transcription by interacting with HMGB DNA-distorting proteins potentially leading to the contortion of DNA within the *CFTR* gene body. The repression of this lncRNA results in derepression of the *CFTR* gene and increased expression of functionally relevant CFTR. The findings reported here not only define a new paradigm for lncRNA regulation of transcription, but also offer insights into a new therapeutically relevant target for bolstering *CFTR* expression to ameliorate CF.

## Results

### Identification of a *CFTR*-associated lncRNA that regulates *CFTR* gene expression

**Figure 1:**
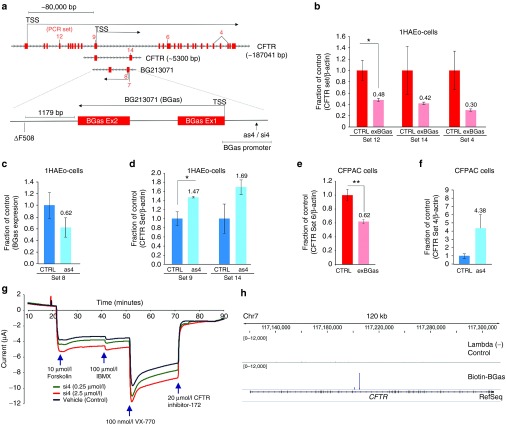
**BGas and as4 mediated regulation of *CFTR*.** (**a**) A schematic depicting the *CFTR* locus with transcriptional start sites (TSS) for *CFTR*, EST BG213071 (BGas), and those primers used to evaluate *CFTR* expression. The sasRNA target site (as4) in the BGas promoter is also shown along with the two exons making up BGas. (**b**) The effects of BGas over-expression using an exogenously expressed BGas (exBGas) on *CFTR* expression in 1HAEo- cells. The control (pcDNA3.1) and exBGas transfected cells are shown. (**c,d**) The effects of sasRNA as4 relative to control (pU6M2) on (**c**) BGas and (**d**) *CFTR* expression in 1HAEo- cells. (**e,f**) The effects of exBGas (**e**) and sasRNA as4 (**f**) on *CFTR* expression in CFPAC cells. (**g**) Dose-dependent effect of an siRNA targeting the as4 site (siRNA4) on ΔF508-*CFTR* chloride channel transport function in CF Human Bronchial Epithelial (CFhBE) primary cells (epithelial voltage clamp assay, Ussing chamber). (**h**) The localization of biotin containing BGas at the intergenic locus of *CFTR* in transfected CFPAC cells contrasted with the lambda biotin control. For **b–f**, the averages of triplicate treated cultures are shown with the standard error of the means and a *P* value from a paired *T*-test, *P < 0.05 and **P < 0.01.

CF is often the result of insufficient CFTR expression on the cell surface. A method capable of bolstering both wildtype and mutant forms of CFTR expression could prove highly useful as a therapeutic strategy for treating CF patients. We therefore sought to investigate the presence of *CFTR*-associated lncRNAs that might function to epigenetically regulate *CFTR* gene expression. Analysis of the *CFTR* locus in the UCSC genome browser revealed an interesting *CFTR*-associated lncRNA, EST BG213071 (**Supplementary Table S1**), that we have designed as BGas. BGas is embedded within the transcribed region, between exons 11 and 12 in the antisense orientation of *CFTR* (**Figure 1a**). Curiously BGas terminates just ~1179bp downstream of the well-known ▵508 mutation and in a region that has been observed previously to exhibit enhancer like properties^15^ (**Figure 1a** and **Supplementary Figure S1**). When BGas was overexpressed in human airway epithelial 1HAEo- cells,^16^ suppression of *CFTR* was observed (**Figure 1b**). Conversely, transcriptional repression of BGas by small antisense RNAs (sasRNAs) (**Supplementary Figure S1a,b**) resulted in significant activation of *CFTR* in 1HAEo- cells (**Figure 1c**,**d**). A similar discordant relationship between BGas and *CFTR* was also observed in CFPAC cells^17^ (**Figure 1e**,**f**), which exhibit similar endogenous levels of BGas expression relative to *CFTR* to those observed in 1HAEo- cells (**Supplementary Figure S1c**). Notably, the activation of *CFTR* by sasRNA as4 resulted in increased CFTR that was functionally viable with regards to CFTR ion transport (**Figure 1g**).

### Mechanism of BGas-mediated *CFTR* regulation

To further investigate the interaction between BGas and *CFTR*, a biotin-labeled BGas transcript was generated and its bound loci in the human genome determined by ChIP-sequencing. The only locus in the entire human genome bound with this biotin-BGas transcript was the BGas locus in intron 11 of *CFTR* (**Figure 1h**). This binding was specifically at the exon 1 of BGas (**Supplementary Figure S2a,b**). Collectively, these data suggest that BGas functions in *cis* and has *CFTR* regulatory properties.

**Figure 2:**
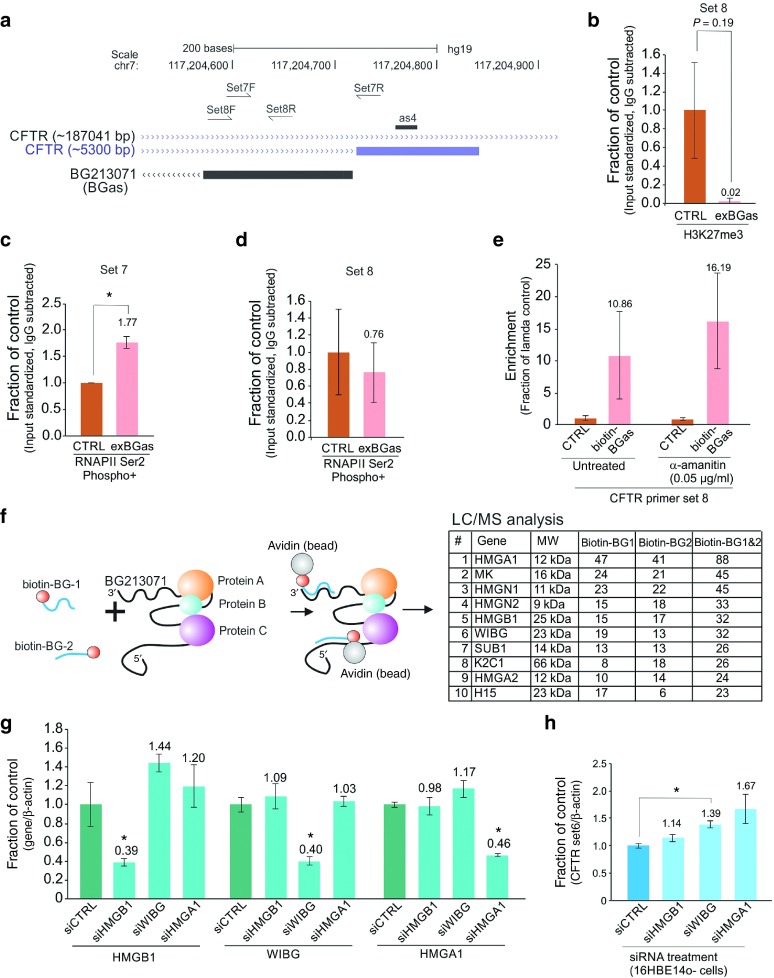
**The mechanism of BGas regulation of *CFTR***. BGas interacts specifically in the *CFTR* locus to modulate the binding of several proteins involved in DNA architecture and chromatin structure. (**a**) A close up snap-shot from UCSC genome browser of the as4 target site and those primers used to distinguish the epigenetic changes at the BGas targeted locus in intron 11 of *CFTR*. (**b**) The effects of over-expression of BGas using an exogenously expressed Bgas (exBGas) relative to control (pcDNA3.1) on H3K27me3 enrichment at BGas exon 1 (Set 8). (**c,d**) Relative enrichment of active RNAPII (serine 2, phospho 5) at the (**c**) BGas exon1/promoter (Set 7) or (**d**) BGas exon 1 (Set 8) in BGas transfected CFPAC cells. (**e**) Transcription is not required for BGas localization to *CFTR*. CFPAC cells were transfected with a biotin-labeled BGas transcript or Lambda transcript (control) (50 nmol/l) and then treated with alpha-amanitin. Biotin pulldowns were carried out 30 hours later to determine localization of the Biotin-BGas transcript. For **b–e**, the averages of triplicate treated cultures are shown with the standard error of the means and a *P* value from a paired *T*-test, *P < 0.05. (**f**) Determination of BGas associated proteins. Biotin-labeled oligonucleotides antisense to BGas (biotin-BG-1 and biotin-BG-2), were used to immunoprecipitate BGas in CFPAC cells. The eluates were subjected to LC/MS analysis and several candidates determined. Total spectrum counts for the top 10 candidates found in both biotin-BG-1 and biotin-BG-2 immunoprecipitations are shown. (**g,h**) Validation of LC/MS identified proteins by RNAi. (**g**) The expression of the mass spectrophotometry identified HMGA1, HMGB1, and WIBG was determined following suppression of each transcript with RNAi relative to a scrambled control siRNA (siCTRL) in non-CF 16HBE14o- cells that express high levels of endogenous *CFTR*.^32^ (**h**) The effects of knockdown of HMGB1, WIBG, and HMGA1 on *CFTR* expression. For (**g–h**), the averages of triplicate transfected 16HBE14o- cells are shown with the standard error of the means and p values from a paired two-sided *T*-test, *P < 0.05.

To determine if BGas regulation of *CFTR* involves targeting epigenetic changes to intron 11 of *CFTR*, we overexpressed BGas in CFPAC cells using a plasmid expressing exogenous BGas (exBGas). A loss of repressive chromatin marks, specifically at exon 1 of BGas, was observed in exBGas treated relative to controls cells (**Figure 2a**,**b**), suggesting that BGas does not recruit epigenetic silencing complexes to this locus. Interestingly, the over-expression of BGas in CFPAC cells resulted in the enrichment of active forms of RNA Polymerase II (RNAPII) specifically at the BGas promoter (**Figure 2c**), but not BGas exon 1 (**Figure 2d**), suggesting that BGas is involved in tethering to distinct local chromatin to affect RNAPII function. Notably, the inhibition of transcription with alpha-amanitin did not appear to affect BGas binding to intron 11 of *CFTR* (**Figure 2e**) and neither did RNAse A nor H treatment (**Supplementary Figure S2c**), suggesting that BGas tethering to this locus is independent of transcription and RNA or DNA binding and functions to locally affect RNAPII function.

### Protein cofactors identified to function in concert with BGas

To explore mechanistically how BGas is modulating *CFTR* expression, we precipitated BGas using biotin labeled oligonucleotides and determined those proteins associated in complex with BGas. Several proteins were found associated with BGas (**Figure 2f** and **Supplementary Table S2**), with a subset including non-histone chromosomal proteins (HMG-14 and HMG-17), high mobility group protein B1 (HMGB1) and partner of Y14 and mago (WIBG) (**Figure 2f**), which notably are DNA binding proteins capable of inducing changes to the local chromatin architecture to affect transcription.^18^ Suppression of HMGA1, HMGB1, and WIBG with RNAi (**Figure 2g**) resulted in significantly increased *CFTR* expression (**Figure 2h**), suggesting that these proteins are involved in modulating *CFTR* expression. Collectively, the observations presented here suggest that BGas functions to modulate *CFTR* expression by tethering various structural and chromatin architectural modifying proteins to intron 11 of *CFTR*.

## Discussion

**Figure 3:**
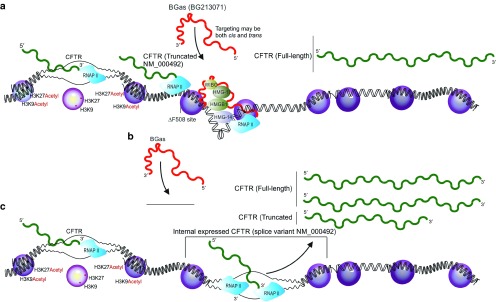
**Model for BG213071 regulation of *CFTR* expression**. (**a**) The *CFTR* locus is shown with the internal expressed BG213071 and truncated *CFTR* transcript NM_000492. (**b**) The BG213071 lncRNA (BGas) is expressed and localizes to the homology-containing locus in the *CFTR* gene body. The localization of BGas to it's target locus allows for chromatin structural and DNA binding proteins such as HMG-14, HMG-17, HMGB1, and WIBG to localize specifically to the *CFTR* gene body and affect the local structure of the gene ultimately diminishing RNAPII activity.

Some antisense lncRNAs have been observed to function as endogenous regulators of epigenetic and transcriptional states of homology containing protein-coding genes (reviewed in refs. 10,11). These antisense transcripts recruit silent state epigenetic marks to particular loci to affect gene transcription. The data presented here suggest that the *CFTR*-associated antisense lncRNA BGas is functionally involved in modulating *CFTR* expression, but in a mechanistically distinct manner that has not been observed previously. BGas appears to function as a scaffolding to partition the *CFTR* locus by possibly tethering chromatin associated proteins such as HMGB1 to a specific region of the gene. HMGB proteins, members of the high mobility group (HMG) superfamily, contain a well-characterized DNA binding domain and have been shown to bind to distorted DNA as well as to induce bending in bound DNA.^19,20^ Recent studies have also shown that HMGBs are able to bind to all immunogenic nucleic acids, which expands their binding affinities to include not only DNA but RNA as well.^21^ Furthermore, Yamanaka *et al*.^22^ recently showed that a natural antisense transcript was implicated in the discordant regulation of low-density lipoprotein receptor-related protein 1 (*Lrp1*) through a mechanism of action that involved the direct binding of the antisense transcript to HMGB2. It is therefore feasible that BGas appears to interact with, and recruit HMGB1 to the *CFTR* locus where HMGB1 may result in the bending and distortion of DNA at specific loci which consequently obstructs RNAPII activity^18^ (**Figure 3**).

BGas tethering or associating with the structural modifying proteins to affect DNA structure and *CFTR* expression is a unique mechanism of transcriptional control embedded in intron 11 of *CFTR* that has not been previously appreciated and may explain previous observation suggesting this region functions as an enhancer.^15,23^ Notably, suppression of BGas and this intronic RNA regulatory system (**Figure 3**) results in substantial increases in *CFTR* expression that may prove therapeutically relevant and suggest that the BGas lncRNA is a bona fide therapeutic target to activate the common ▵F508 variants of *CFTR*.

## Materials and Methods

***Generation of BGas promoter targeted small antisense RNAs and exBGas.*** To delineate any role that EST BG213071 (BGas) might be playing in the regulation of *CFTR*, we generated several small antisense noncoding RNAs (sasRNAs) targeted to the upstream putative promoter sites for BGas. The sasRNA target sites are shown (**Supplementary Figure S1**) and were derived using an algorithm for promoter targeting with sasRNAs.^24^ Vectors expressing the sasRNAs were generated by annealing oligonucleotides (IDT technologies, Coralville, IA) (**Supplementary Table S3**) and subsequent cloning into the pU6M2 construct using the Bgl II and Kpn I restriction sites (as described in ref. 25). Positive clones were determined by sequencing and then midi-prepped (Qiagen, Valencia CA). The control for all sasRNA-transfected cells was the parental plasmid pU6M2. exBGas was commercially synthesized (Genewiz, South Plainfield, NJ) by cloning the BGas sequence (**Supplementary Table S1**) into pcDNA3.1 to generate exBGas. In this manner, BGas is expressed off a CMV promoter or can be *in vitro* transcribed from the T7 promoter.

***Cell culture and transfections.*** CFPAC cells (ATCC Number CRL-1918) (Genotype: CFTR ▵F508/▵F508 (CF)) were maintained in Iscove's Modified Dulbecco Minimum Essential Medium (IDMEM) (Mediatech, Manassas, VA) supplemented with 10% fetal bovine serum (Life Technologies, Carlsbad, CA), 50 µg/ml Pen/Strep (Mediatech) and 1% nonessential amino acids (Mediatech) at 37 °C and 5% CO2. The 1HAEo- airway epithelial cells (genotype: CFTR wt/wt (normal)), 16HBE14o- bronchial epithelial cells (genotype: CFTR wt/wt (normal)) and CFBE41o- bronchial epithelial cells (genotype: CFTR ▵F508/▵F508 (CF)) (a gift from Dieter Gruenert) were maintained in MEM (Life Technologies) supplemented with 10% fetal bovine serum (Life Technologies), 50ug/ml Pen/Strep (Mediatech) and 1% L-Glutamine (Mediatech) at 37 °C and 5% CO2. For transfection, cells were plated in 24-well plates (1–2 × 10^5^ cells/well). Twenty-four hours later, the cultures were transfected using either the Neon electroporation system or Lipofectamine 2000 (Life Technologies), with 100–200 ng DNA.

***qRT-PCR and directional RT analysis of gene expression.*** To determine the BGas targeted sasRNA effects on *CFTR*, CFPAC or 1HAEo- cells were transfected as described above. Total RNA was isolated 72 hours post-transfection using the Maxwell 16 LEV simplyRNA purification kit and the Maxwell 16 Research Instrument (Promega, Madison, WI). DNase-treated RNA samples were then standardized and reverse transcribed with Mu-MLV (Life Technologies) using an oligo-dT/random nonamer primer mix or with strand-specific primers. Quantitative real-time polyemerase chain reaction (qRT-PCR) was carried out using Kapa Sybr Fast universal qPCR mix (Kapa Biosystems, Wilmington, MA) on an Eppendorf Mastercycler realplex. Thermal cycling parameters started with 3 minutes at 95 °C, followed by 40 cycles of 95 °C for 3 seconds and 60 °C for 30 seconds. Specificity of the PCR products was verified by melting curve analysis. Various primer sets were used for qRT-PCR analysis (**Supplementary Figure S1a**, **Supplementary Table S3**). To determine changes in BGas expression, directional RT was carried out using primer Set8F or without any primer (background control). Following the RT step, qRT-PCR was carried out with primer Set8F and Set8R (**Supplementary Figure S1** and **Supplementary Table S3**). For analysis of other genes, random RT primed cDNAs were assessed for particular gene expression relative to β-actin using various locus-specific primers (**Supplementary Figure S1** and **Supplementary Table S3**).

***Chromatin immunoprecipitation.*** ChIP analysis was carried out on the exBGas transfected CFPAC cells (~4 × 10^6^ cells) for suppressive Histone 3 Lysine 27 trimethyl-marks (H3K27me3) (Abcam, ab6002, Cambridge, MA) and active forms of RNA Polymerase II (RNAPII) using anti-anti-RNAPII phospho-S2 (AbCam #ab5095). The ChIP was performed 72 hours post-transfection following previously described techniques.^14,26^ The relative enrichment of the various epigenetic marks was determined at the *CFTR* promoter using primer sets 7 and 8 (**Supplementary Table S3**). IgG or no antibody values were subtracted from the resultant IP and input values and standardized to input.

***T7-transcribed synthetic RNA pulldown for localization studies.*** Synthetic biotinylated ncRNAs were generated by T7 transcription using the Ampliscribe T7-Flash Biotin—RNA Transcription Kit (Epicentre Biotechnologies, Madison, WI) according to the manufacturer's instruction. Templates for T7 transcription was prepared by PCR of pcDNA3.1 plasmids expressing the relevant ncRNAs. The following primers were used: T7-BG213071 F: 5′-CAGTGAATTGTAATACGACTCACTATAGGGGTAATATATCTA-3′ and BG213071 R: 5′-CTCAAAGAGGATATACTTCATTCCTCAAAAGG-3′; T7-AI805#2 F: 5′-CAGTGAATTGTAATACGACTCACTATAGGGCTTTTCTCCGAC-3′ and AI805#2 R: GCTTCCAATTCCCCCCACC; T7-AI805#1 F: 5′-CAGTGAATTGTAATACGACTCACTATAGGGTCGGAGAAAAGA-3′ and T7-AI805#1 R: 5′-GAAGGCGCCTACGCCTG-3′. Transcripts were transfected into CFPAC cells at a concentration of 50 nmol/l. Thirty hours post-transfection, cells were cross-linked with formaldehyde at 1% for 10 minutes at room temperature followed by addition of glycine to a final concentration of 0.125M and a further incubation for 5 minutes at room temperature. Cells were then washed with phosphate buffered solution (PBS) supplemented with PMSF, aproteinin and leupeptin and lysed with ChIP lysis buffer (50 mmol/l Hepes, 140 mmol/l NaCl, 1% Triton X, 0.1% NAD) on ice for 20 minutes. Chromatin was sheared by sonication. Cell lysates containing sheared chromatin, or ChIP eluates in the case of ChIP-biotin dual pull-down assays, were incubated with Dynabeads MyOne Streptavidin C1 (Life Technologies) prepared according to the manufacturer's instructions for 2 hours on a rotating platform. Beads were pulled down with a magnet for 3 minutes and washed with low-salt immune complex wash buffer (0.1% SDS; 1% Triton X-100; 2 mmol/l Ethylenediaminetetraacetic acid (EDTA); 20 mmol/l Tris-HCl, pH 8.1; 150 mmol/l NaCl); High-salt immune complex wash buffer (0.1% SDS; 1% Triton X-100; 2 mmol/l EDTA; 20 mmol/l Tris-HCl, pH 8.1; 500 mmol/l NaCl); LiCl Immune complex wash buffer (0.25 M LiCl; 1% NP40; 1% sodium deoxycholate; 1 mmol/l EDTA; 10 mmol/l Tris-HCl, pH 8.1); and TE buffer (10 mmol/l Tris-HCl; 1 mmol/l EDTA, pH 8.0). Each wash step was carried out for 3 minutes on a rotating platform. Streptavidin bead-biotinylated RNA-DNA complexes were resuspended in nuclease-free water and heated at 95 °C for 5 minutes to denature RNA-DNA hybrids. Streptavidin bead-biotinylated RNA complexes were pulled down with a magnet and DNA-containing supernatants were analyzed by qPCR. For RNase A and RNase H treatments, streptavidin bead-biotinylated RNA-DNA complexes were exposed to RNAse A (10 μg/ml) or RNase H (2 units) (Thermo Fisher Scientific, Waltham, MA) after the final wash step for 30 minutes at 37 °C. The samples were then heat inactivated (95 °C for 10 minutes for RNase A and 65 °C for 20 minutes for RNase H) and prepared for qPCR as described above.

***Biotin-tagged oligo pull down for mass spectrometry studies.*** CFPAC cells were cross-linked with formaldehyde at 1% for 10 minutes at room temperature followed by addition of glycine to a final concentration of 0.125M and a further incubation for 5 minutes at room temperature. Cells were then washed with and resuspended in PBS supplemented with PMSF, aproteinin and leupeptin and lysed with ChIP lysis buffer (5 mmol/l PIPES, 85 mmol/l KCl, 0.5% NP40) on ice for 20 minutes. Cell lysates were incubated with the following biotin-tagged oligos: biotin-BG-1: 5′-/5Biosg/GCCAGCACAAGAATCCCTCA-3′ and biotin-BG-2: 5′-/5Biosg/CCAAATGCAAACATTCATGATTC-3′ for 15 minutes on a rotating platform at room temperature. Cell lysate/biotin-tagged oligo solutions were incubated with Dynabeads MyOne Streptavidin C1 (Life Technologies) prepared according to the manufacturer's instructions for 15 minutes on a rotating platform. Captured compounds were pulled down with a magnet for 3 minutes and washed twice with PBS supplemented with PMSF, aproteinin and leupeptin. Each wash step was carried out for 3 minutes on a rotating platform. Proteins were eluted with 50 µl elution buffer (10 mmol/l Tris-HCl (pH 6.0), 1 mmol/l EDTA, and 2.0 M NaCl) at 70 °C for 10 minutes. The eluates were used for mass spectrometry.

***Liquid chromatography mass spectrometry analysis (LC/MS).*** Those elutes from the biotin tagged oligonucleotide IP (described above) were subjected to mass spectrometry analysis (BMSF core facility at the University of New South Wales, Australia). Nano-Liquid chromatography (nano-LC) was performed using an Ultimate 3000 HPLC and autosampler system (Dionex, Amsterdam, Netherlands). Samples were injected into a fritless nanoLC column (75 µm × ~10 cm) containing C18 media (3 µm, 200 Å Magic, Michrom) manufactured according to Gatlin.^27^ Peptides were eluted using a linear gradient according to the conditions in the table below, over 50 minutes, at a flow rate of 0.2 µl/minute. Mobile phase A consisted of 0.1% formic acid in H_2_O, while mobile phase B consisted of ACN:H_2_O (8:2) with 0.1% formic acid. Tandem mass spectrometry (MS/MS): Electrospray tandem mass spectrometry was performed with the LTQ Fourier transform ion cyclotron resistance mass spectrometer (Thermo Fisher Scientific). High voltage (1,800 V) was applied to a low volume tee (Upchurch Scientific) and the column tip positioned ~0.5 cm from the heated capillary (T = 250 °C) of a LTQ FT Ultra (Thermo Electron, Bremen, Germany) mass spectrometer. Positive ions were generated by electrospray and the LTQ FT Ultra operated in data-dependent acquisition mode. A survey scan m/z 350–1,750 was acquired in the FT ICR cell (resolution = 100,000 at m/z 400, with an accumulation target value of 1,000,000 ions). Up to the six most abundant ions (>3,000 counts) with charge states > +2 were sequentially isolated and fragmented within the linear ion trap using collisionally induced dissociation with an activation *q* = 0.25 and activation time of 30 ms at a target value of 30,000 ions. M/z ratios selected for MS/MS were dynamically excluded for 30 seconds.

***Analysis of mass spectrometry database search parameters.*** Peak lists were generated using Mascot Daemon/extract_msn (Matrix Science, London, England, Thermo) using the default parameters, and submitted to the database search program Mascot (Matrix Science, London, UK; version 2.3.02) (Perkins, D et al Electrophoresis 1999:20:3551–3567) (ref. 28). Mascot was used to search the UniProtKB/Swiss-Prot database (released 25 October 2013) containing 20,352 sequence entries with the database taxonomy restricted to Homo sapiens. The peptide mass tolerance and fragment ion mass tolerance were set to 4 ppm and ± 0.4 Da respectively. Trypsin was specified as the enzyme in Mascot with allowance for up to one missed cleavage site per peptide. Oxidation of methionine and carbamidomethylation of cysteine were set as variable modifications within Mascot.

***Quantification and validation of protein identifications.*** Scaffold (version Scaffold_4.3.2, Proteome Software, Portland, OR) was used to validate MS/MS-based peptide and protein identifications from Mascot and to compare the relative spectral counts between the biotin-BG-1 and biotin-BG-2 IP samples. The mass spectra from triplicate biological pull-down/LC/MS experiments were combined to give two biological samples: biotin-BG-1 and biotin-BG-2. Peptide identifications were accepted if they could be established at greater than 95.0% probability by the Peptide Prophet algorithm^29^ with Scaffold delta-mass correction. Protein identifications were accepted if they could be established at greater than 99.9% probability by the Protein Prophet algorithm^30^ and contained at least 3 unique identified peptides. A total of 1,850 spectra at 95% minimum probability (0.42% peptide false discovery rate) identified 44 proteins containing at least three unique peptides at 99.9% probability by the Peptide Prophet algorithm (at 0.0% protein false discovery rate). Total spectral counts for the biotin-BG-1 and biotin-BG-2 pooled biological samples were compiled in Scaffold_4.3.2 and the proteins were identified (**Supplementary Table S2**).

***Suppression of BG213071-associated proteins.*** Those protein candidates determined by LC/MS of biotin-BG213071 eluates were suppressed using RNAi. Commercially available siRNAs for HMGA1 (siRNA sc-44333, Santa Cruz, Santa Cruz, CA), HMGN2 (siRNA sc-37988, Santa Cruz), HMGB1 (siRNA sc-37982, Santa Cruz), WIBG (siRNA sc-96076, Santa Cruz) and a scrambled control (siRNA sc- 37007, Santa Cruz) were transfected into 1HAEo- CFPAC, or 16HBEo- cells (50nM) using the Neon Electroporator (Life Technologies). The transfected cultures were collected 72 hours later and gene expression determined using qRT-PCR as described above.

***Efficacy of test articles on ΔF508-CFTR chloride channel transport function in monolayers of cystic fibrosis human bronchial epithelial primary culture cells.*** Ussing epithelial voltage clamp assay was performed according to the method of.^31^ Briefly, primary human bronchial epithelial (hBE) cells from a CF patient homozygous for ΔF508-*CFTR* (Asterand Biosciences, Royston, UK) were plated onto Costar Snapwell (Corning, Corning, NY) tissue-culture inserts coated with collagen type IV, and cultured in Bronchial epithelial growth medium (Lonza, Basel, Switzerland). An siRNA targeted to the as4 site (siRNA4) was mixed with MessengerMax (Life Technologies) according to manufacturer's instruction, and added to the top surface (apical) of the epithelia at the bottom of the cup of the Snapwell insert. Four hours after transfection, culture medium containing the transfection mixture was replaced with fresh medium and incubated at 37 °C overnight. The hBE cell monolayers grown on the filter inserts were transferred to Physiologic Instruments Ussing recording chambers (Physiologic Instruments, San Diego, CA) and superfused in both the apical and basolateral chambers with a physiological saline (pH 7.4) containing 137 mmol/l NaCl, 4 mmol/l KCl, 1.8 mmol/l CaCl_2_, 1 mmol/l MgCl_2_, 10 mmol/l HEPES, and 10 mmol/l glucose. Chloride transport function of the cell was monitored as the *CFTR* agonist evoked short circuit (ISC) current output of an Ussing chambers and an epithelial voltage clamp using 6-channel Physiologic Instruments VCC MC6 or VCC MC8 epithelial voltage clamps (Physiologic Instruments). Data acquisition and analyses were performed using iWorx data acquisition hardware and Labscribe 2 software (iWorx, Dover, NH).

**SUPPLEMENTARY MATERIAL** **Figure S1.**The *CFTR* locus of interest including reported antisense transcript BG213071. **Figure S2.** Biotin-labeled BGas localization in *CFTR*. **Table S1.** Sequence of EST BG213071 (BGas) cloned into pcDNA3.1. **Table S2.** Mass spec identified proteins associated with BGas. **Table S3.** Oligonucleotide and primer sequences.
