# Supplementary material for: Long Non-coding RNA BGas Regulates the Cystic Fibrosis Transmembrane Conductance Regulator
Source: Mol Ther. 2016 Jul 19;24(8):1351–7. doi: 10.1038/mt.2016.112 (PMC5023374; doi:10.1038/mt.2016.112)
Supplement: Supplementary Figures and Tables [file mt2016112x1.doc]

**Supplemental Information**

**Long Non-coding RNA BGas Regulates the Cystic Fibrosis Transmembrance Conductance Regulator**

**
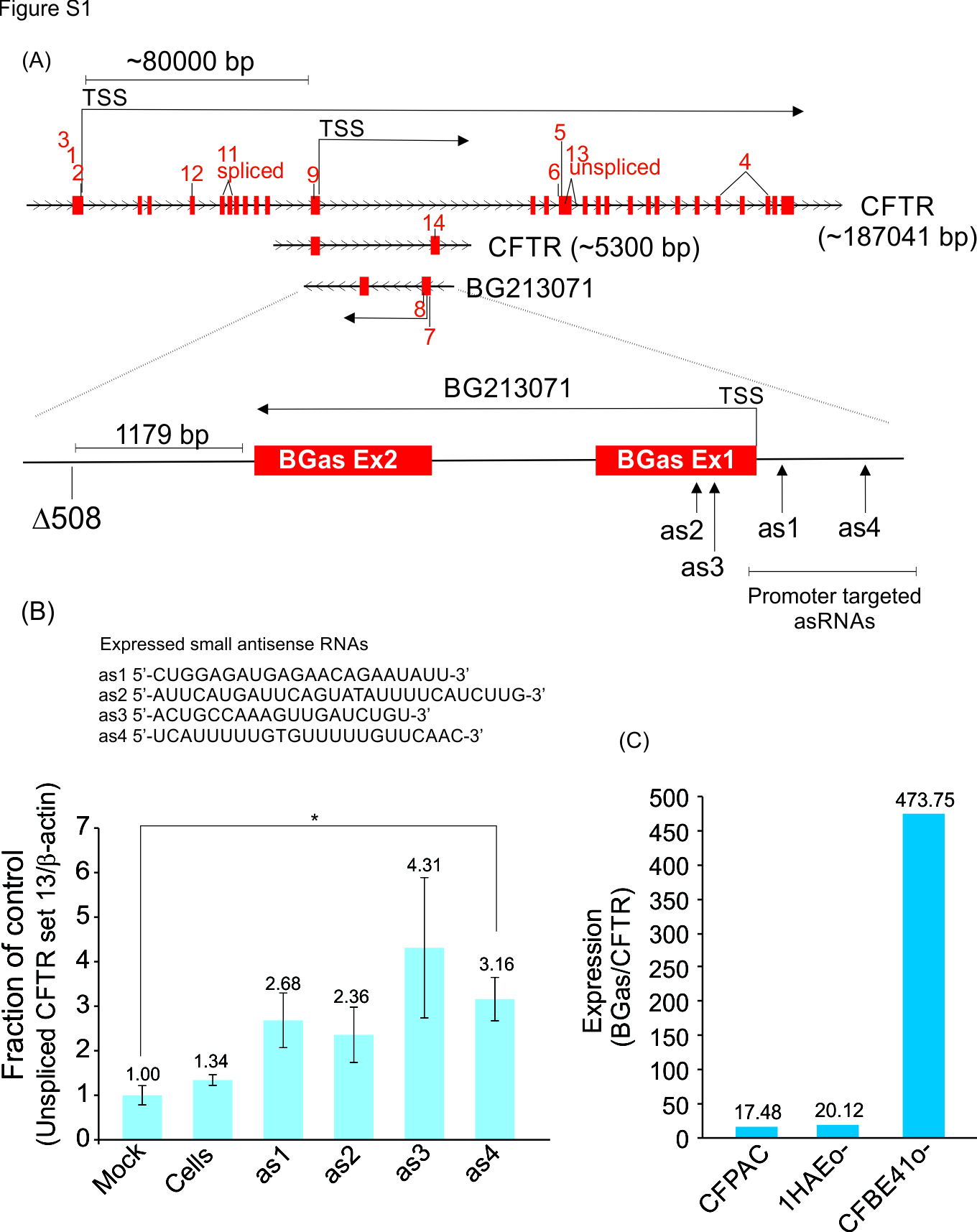
**

**Figure S1.** **The *CFTR* locus of interest including reported antisense transcript BG213071.**

(A) The transcriptional start sites (TSS) of *CFTR* and BG213071 (BGas), are shown along with the various primer binding sites and small antisense RNA target sites in *CFTR* (as1-4). (B) The sequences of the expressed small antisense RNAs targeted to the BGas promoter (inset) and the effects of the small antisense RNAs targeting BGas on *CFTR* expression. The small antisense RNA expressing plasmids (as1-4) or control pU6M2 (Mock) were transfected in triplicate into CFPAC cells and unspliced *CFTR* mRNA assessed. *CFTR* expression was measured 72hrs later and the average of triplicate treated CFPAC cells is shown with the standard error of the means and p values from a paired T-test *p<0.05. (C) The expression of BGas was determined by strand specific RT (using the *CFTR* Set 8F primer) and PCR using primer set 8. No primer RT controls were subtracted. The expression of *CFTR* was determined by RT with a primer mix followed by PCR with primer set 8 and no RT controls were subtracted. The ratio of BGas to *CFTR* was then determined for each cell line.


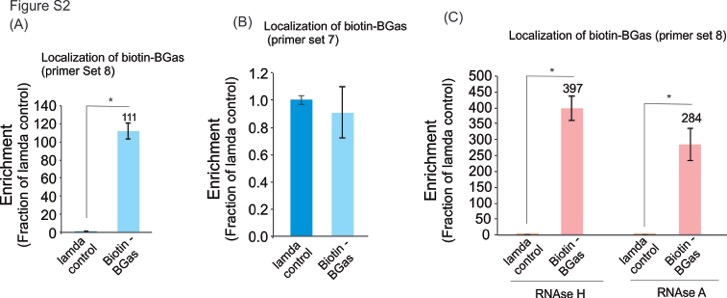
**Figure S2. Biotin-labeled BGas localization in *CFTR*.**

The localization of biotin-labeled BGas at the (A) BGas Exon 1 or (B) BGas promoter in CFPAC cells. (C) Effects of RNAse H and RNAse A treatment on biotin-labeled BGas localization. For A-C the averages of triplicate treated cultures are shown with the standard error of the means and (*) indicating p value from a paired T-test <0.001.

**Table S1. Sequence of EST BG213071 (BGas) cloned into pcDNA3.1.**

5’GTAATATATCTAAAAAACACATCAACTTTGGCAGTCAAAATGAAAATATACTGAATCATGAATGTTTGCATTTGGTGATCGGGGCTTCATAGAGAACATATTGAGGGATTCTTGTGCTGGCACCAGCTGCTTAACCATCAAGGATTCTGTTATGAAATACTATTCCTGGAAGAAGGCCTTTTGAGGAATGAAGTATATCCTCTTTGAG3’

**Table S2.** **Mass spec identified proteins associated with BGas.**

The molecular weight of the proteins is shown (MW) along with the % coverage from each pull-down with 5’ biotin linked oligonucleotides biotin-BG-1 or biotin-BG-2.


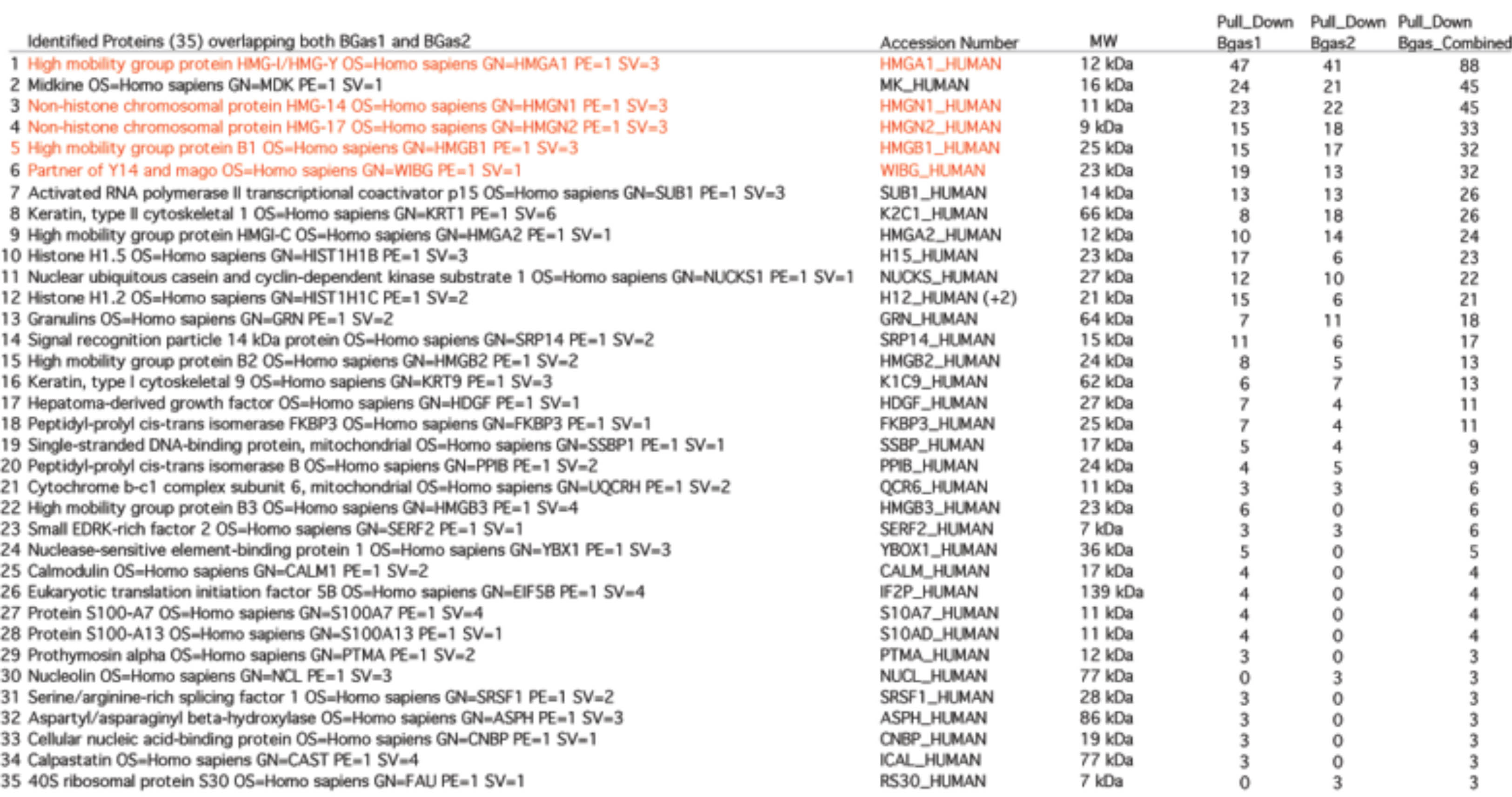


**Table S3.** Oligonucleotide and primer sequences.

| Name | Sequence (5’-3’) | Function |
| --- | --- | --- |
| Set 1F | TGCAAACGTAACAGGAACCCGACT | *CFTR* locus specific |
| Set 1R | TCTTTAGGTCCAGTTGGCAACGCT | *CFTR* locus specific |
| Set 2 F | GGAAGCAAATGACATCACAGCAGG | *CFTR* locus specific |
| Set 2R | TGGGCTCAAGCTCCTAATGCCAAA | *CFTR* locus specific |
| Set 3F | AAGACTGCTTGGCTTCCTTTCGGT | *CFTR* locus specific |
| Set 3R | AATGCTTTGCGTAATTACCGGCCC | *CFTR* locus specific |
| Set 4F | GTGGAGGAAAGCCTTTGGAGT | *CFTR* locus specific |
| Set 4R | ACAGATCTGAGCCCAACCTCA | *CFTR* locus specific |
| Set 5F | AGCCTTTAGAGAGAAGGCTGTCCT | *CFTR* locus specific |
| Set 5R | GTGTGGATGCTGTTGTCTTTCGGT | *CFTR* locus specific |
| Set 6F | CTCATGGGATGTGATTCTTTCGACC | *CFTR* locus specific |
| Set 6R | TTTCTGTCCAGGAGACAGGAGCAT | *CFTR* locus specific |
| Set 7F | GTGCCAGCACAAGAATCCCTCAAT | Directional RT for BG213071 expression |
| Set 7R | TGTTCTCATCTCCAGTTCCAAGTC | Directional RT for BG213071 expression |
| Set 8F | TTGATGGTTAAGCAGCTGGTGCC | Directional RT for BG213071 expression |
| Set 8R | GAATGTTTGCATTTGGTGATCGGG | Directional RT for BG213071 expression |
| Set 9F | GGTGATTATGGGAGAACTGGAG | Internal *CFTR* promoter (Exon11/intron 11, BGas regulated?) |
| Set 9R | TCTTTAATGGTGCCAGGCATA | Internal *CFTR* promoter (Exon11/intron 11, BGas regulated?) |
| Set 10F | TTTGGACTTACCTCAAAGAGGAT | Internal *CFTR* promoter (Exon11/intron 11, BGas regulated?) |
| Set 10R | TCCAACCTCCAGGTTATGAAAT | Internal *CFTR* promoter (Exon11/intron 11, BGas regulated?) |
| Set 11F | CCTTTCCAACAACCTGAACAAA | *CFTR* Exon 5/6 splicing |
| Set 11R | GCCTGTAACAACTCCCAGATTA | *CFTR* Exon 5/6 splicing |
| Set 12F | GCTTCCTATGACCCGGATAAC | *CFTR* Exon 4 |
| Set 12R | GGAGCAGTGTCCTCACAATAA | *CFTR* Exon 4 |
| Set 13F | CAGGCAAACTTGACTGAACTGG | *CFTR*_unspliced |
| Set 13R | GCATTCTACTCAATTGCATTCTGTGGG | *CFTR*_unspliced |
| Set14F | TTGTTCAACTCTAATGTCTGCAAAG | *CFTR*_internal Spliced variant |
| Set14R | CACTTACCTGCCTGCTCCTA | *CFTR*_internal Spliced variant |
| biotin-BG-1 | GCCAGCACAAGAATCCCTCA | 5’Biotin oligonucleotides for IP of BGas |
| biotin-BG-2 | CCAAATGCAAACATTCATGATTC | 5’Biotin oligonucleotides for IP of BGas |
| HMGA1_F1 | GAGGAAGAGGAGGGCATCTcg | *HMGA1* mRNA detection |
| HMGA1_R | TGTCCAGTCCCAGAAGGAAgc | *HMGA1* mRNA detection |
| HMGN1_F1 | CTCTGATGAAGCAGGAGAGAAAG | *HMGN1* mRNA detection |
| HMGN1_R1 | AGACAGGGACCACTGATAAGA | *HMGN1* mRNA detection |
| HMGN1_F2 | GGCAGCAGCGAAGGATAAAt | *HMGN1* mRNA detection |
| HMGN1_R2 | CCGCAGGTAAGTCTTCTTTAGTT | *HMGN1* mRNA detection |
| HMGN2_F1 | GGAATGCTGCCTCTGATCTT | *HMGN2* mRNA detection |
| HMGN2_R | ATGGAGTACCTCAAAGCAGAAC | *HMGN2* mRNA detection |
| HMGB1_F1 | TCATAAGGCTGCTTGTCATCT | *HMGB1* mRNA detection |
| HMGB1_R1 | GCTCTGAGTATCGCCCAAAaa | *HMGB1* mRNA detection |
| HMGB1_F2 | CCACATCTCTCCCAGTTTCTTC | *HMGB1* mRNA detection |
| HMGB1_R2 | GTTCGGCCTTCTTCCTCTTC | *HMGB1* mRNA detection |
| WIBG_F1 | CGGAGGGTGAAAGAAGGATATG | *WIBG* mRNA detection |
| WIBG_R1 | GGGCAACTCTGGTTTACTCTT | *WIBG* mRNA detection |
